# The genetic legacy of fragmentation and overexploitation in the threatened medicinal African pepper-bark tree, *Warburgia salutaris*

**DOI:** 10.1038/s41598-020-76654-6

**Published:** 2020-11-12

**Authors:** Annae M. Senkoro, Pedro Talhinhas, Fernanda Simões, Paula Batista-Santos, Charlie M. Shackleton, Robert A. Voeks, Isabel Marques, Ana I. Ribeiro-Barros

**Affiliations:** 1 Department of Environmental Science, Rhodes University, Grahamstown, 6140 South Africa; 2 Departmento de Ciências Biológicas, Universidade Eduardo Mondlane CP 257, Maputo, Moçambique; 3 Linking Landscape, Environment, Agriculture and Food (LEAF), Instituto Superior de Agronomia, Universidade de Lisboa, Tapada da Ajuda, 1349-017 Lisbon, Portugal; 4 Instituto Nacional de Investigação Agrária E Veterinária, Av. da República, Quinta Marquês, Edificio Sede, 2780-157 Oeiras, Portugal; 5 Department of Geography and the Environment, California State University, 800 N State College Blvd, FullertonFullerton, CA 92831 USA; 6 Forest Research Centre (CEF), Instituto Superior de Agronomia, Universidade de Lisboa, Tapada da Ajuda, 1349-017 Lisbon, Portugal

**Keywords:** Molecular biology, Plant sciences

## Abstract

The pepper-bark tree (*Warburgia salutaris*) is one of the most highly valued medicinal plant species worldwide. Native to southern Africa, this species has been extensively harvested for the bark, which is widely used in traditional health practices. Illegal harvesting coupled with habitat degradation has contributed to fragmentation of populations and a severe decline in its distribution. Even though the species is included in the IUCN Red List as *Endangered*, genetic data that would help conservation efforts and future re-introductions are absent. We therefore developed new molecular markers to understand patterns of genetic diversity, structure, and gene flow of *W. salutaris* in one of its most important areas of occurrence (Mozambique). In this study, we have shown that, despite fragmentation and overexploitation, this species maintains a relatively high level of genetic diversity supporting the existence of random mating. Two genetic groups were found corresponding to the northern and southern locations. Our study suggests that, if local extinctions occurred in Mozambique, the pepper-bark tree persisted in sufficient numbers to retain a large proportion of genetic diversity. Management plans should concentrate on maintaining this high level of genetic variability through both *in* and *ex-situ* conservation actions.

## Introduction

Medicinal plants have been used worldwide since ancient times, being particularly relevant in the developing world where *ca.* 80% of the population rely on these resources to fulfil their basic health care needs^1–4^. Additionally, at the global level the importance of bio-based compounds continues to grow and phytochemical research towards the identification of new active compounds of medical and nutritional importance is among top research priorities (e.g.^5–14^).

Sub-Saharan Africa harbours a vast repository of plant biodiversity, with 45,000 known vascular plant species^15^, many of which are used in traditional medicine^16–20^. However, efforts to safeguard this biodiversity are often compromised by anthropogenic pressures, with proximal drivers being land transformation, synergistic impacts of fires, grazing, climate change and harvesting (c.f.^17,21–27^), and growing commercialisation of medicinal plant in high demand (c.f.^17,28,29^). The last is motivated by preferences for certain species due to cultural identity, traditions, and lower costs in comparison with modern pharmaceuticals, even under circumstances of access to modern medical facilities^21,30^. On the other hand, the conservation status of many endemic and native species is poorly understood^31,32^ and many natural populations may be at risk. Current exploitation rates, often in tandem with other pressures like fire, invasive species, browsing and land transformation, threaten wild populations unless management methods are established, including community-based approaches^17,21,30^.

Under the current scenario of climate change and human population growth, the use of genomic tools is valuable to understand species evolution and adaptation in natural ecosystems^33,34^. The importance of phylogenetic data, genetic diversity, and population structure analyses to characterize the biodiversity of wild species has been well-established in numerous studies (e.g.^35–39^). Microsatellites (Single Sequence Repeats, SSR) are amongst the most efficient and widely used markers for these studies as they are codominant and highly polymorphic loci^40^. Although these markers are species specific, the increasing accessibility to next-generation sequencing^41^ has enabled the development of SSRs for the so-called orphan, neglected or wild crop relative species (e.g.^42–45^), although sequencing large plant genomes still remains a challenge^46^.

The pepper-bark tree, *Warburgia salutaris* (Bertol.) Chiov. (Family Canellaceae) is one of the most widely used and traded medicinal plants in southern Africa^47^. This slow growing species is part of an early diverging group of basal angiosperms, thought to be native to eastern and southern Africa^48^. However, subsequent studies confined the distribution of *W. salutaris* to only a sub-region of southern Africa, *i.e.* South Africa^21,49^, Eswatini (previously known as Swaziland)^24,50^, Zimbabwe^29,51–53^, Malawi^54^ and Mozambique^55^. This species is commonly used to treat several ailments such as common colds, throat and mouth sores, or coughs^47,48^.

**Figure 1:**
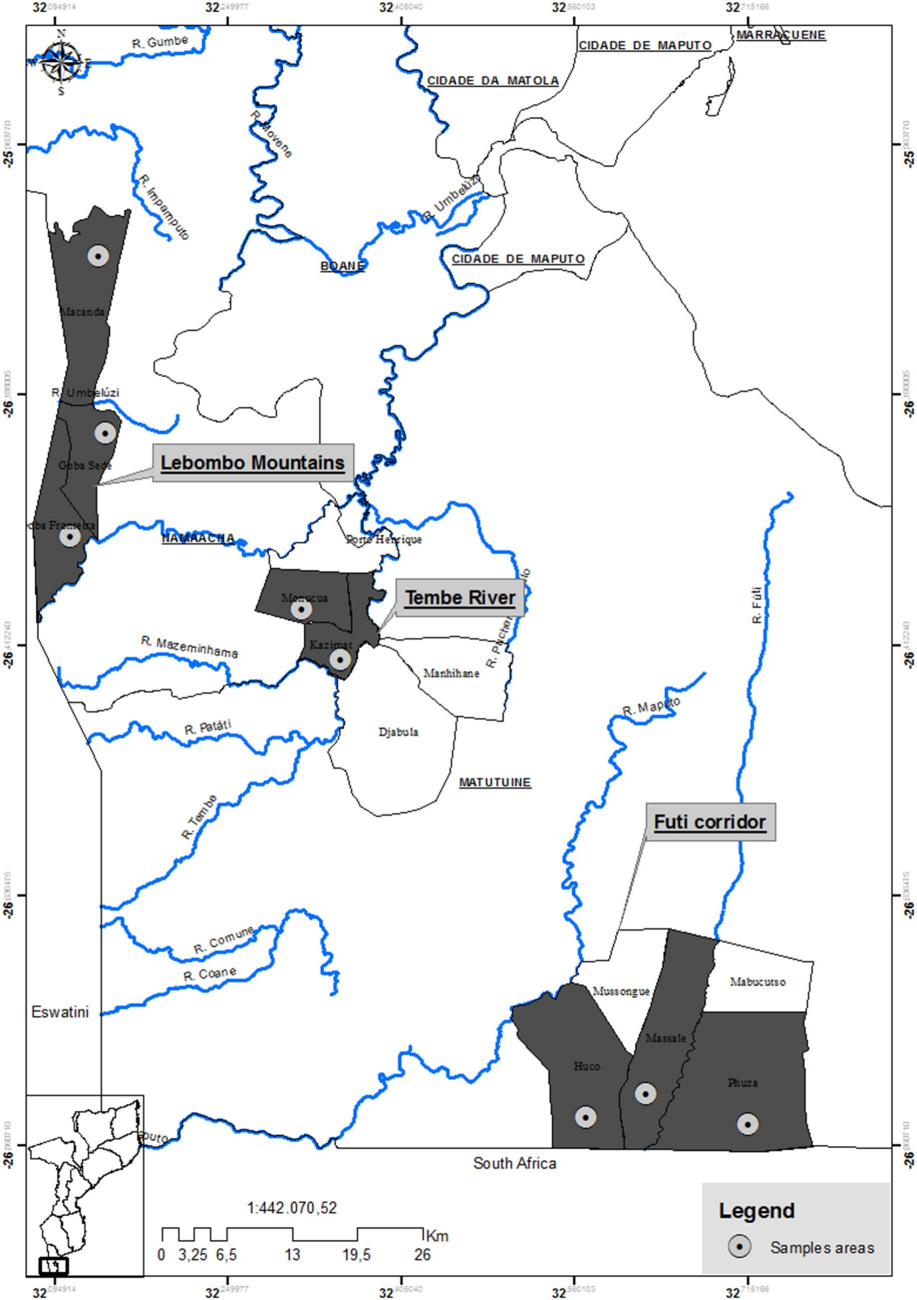
Location of the Lebombo Mountains, Tembe River, and Futi Corridor areas and their respective villages in southern Mozambique. Maps were generated with Idrisi Selva v.17.02 environment (Clark Labs, Clark University, www.clarklabs.org).

In the past, sustainable harvesting of medicinal plants was regulated through traditional practises such as taboos, restrictions and harvesting tools^30^. However, with commercial demand increasing, *W. salutaris* groves were repeatedly raided by harvesters that often debarked the whole tree, especially mature plants^56^. Severe harvesting resulted in high tree mortality in many areas and in the extinction of many local populations^21,24,57^ and consequently, *W. salutaris* is considered threatened throughout its range^50,58,59^, and listed as an *Endangered Species* in the IUCN Red List^57^. The most extreme case is that of Zimbabwe, where the species is listed as extinct in the wild^29,60^. That resulted in the import of bark supplies in the late 1990s from Mozambique and South Africa^53^ being later trafficked from the same countries^29^. For instance, in South Africa, 43% of *W. salutaris* bark in the Johannesburg main market originated from Mozambique, with annual traded amounts estimated at 500–1000 kg^28^. As a result, populations of *W. salutaris* in Mozambique are currently restricted to fragmented patches in the Lebombo Mountains, Tembe River and Futi Corridor (Fig. 1)^48^. According to the Red List classification for Mozambique, this species is considered *Vulnerable VU A2 cd*^58^. Despite this critical situation, only a few studies on the populations dynamics of *W. salutaris* are available; of the 60 research and review papers available in the Web of Science on *W. salutaris* on 05 February 2020, only seven addressed this topic^21,24,48,61–63^ while the vast majority are focused on the medicinal applications of this species. Nevertheless, amplified fragment length polymorphisms (AFLPs) have been used to solve genetic relationships between *W. ugandensis*, *W. salutaris* and *W. stuhlmanni* showing a high degree of genetic variation among individuals within populations as well as between populations^62^.

In this work, we have developed SSRs markers for *W. salutaris* to investigate the genetic legacy of exploitation in this slow growing species and to contribute to future re-introduction actions. For that, we have used its best known area of occurrence, Mozambique (Fig. 1) to addressed the following questions: (1) How is genetic diversity distributed within and among individuals across geographical areas?; (2) Is the genetic structure associated with the geographical distribution?; and (3) Is there any evidence of inbreeding or lack of gene flow between populations?

## Results

### Genetic diversity

For each locus*,* the numbers of alleles varied from three (13-N1132836, 16-N1150626 and 18-N1173706 locus) to nine (31-N2284857 and 43-N1009973 locus) with an average of 5.8 ± 2.3 alleles per locus and a total of 58 alleles considering all loci (Table 1). The average observed and expected heterozygosis per loci varied from 0.299 ± 0.186 (16-N1150626) to 0.852 ± 0.062 (10-N1110523), and from 0.249 ± 0.109 (16-N1150626) to 0.812 ± 0.048 (31-N2284857), respectively.

**Table 1:** Characteristics and genetic diversity statistics of the 10 polymorphic microsatellite markers developed for *Warburgia salutaris.*

Locus	Repeat motif	Accession number	Primer Sequence 5′–3′	Size range	*Na*	*Ho*	*He*
1-N1002135	(ATG)5	MT515706	F: TATGTTGGGAGAGGGTGAGG R: GTTTAACGACTGCATCATCCCA	132–174	6	0.487 ± 0.139	0.394 ± 0.101
7-N1082598	(AAT)9	MT515707	F: GTTGATCATAGACACGCCAAGG R: GTCGTGCAACCTAGAGGTCC	161–182	7	0.633 ± 0.085	0.700 ± 0.029
10-N1110523	(TTA)9	MT515708	F: AACCATTGGCACCTCAAGTC R: GTTGAAGTTGAGGGAAGGGATG	244–262	7	0.852 ± 0.060	0.786 ± 0.023
12-N1126672	(TTG)7	MT515709	F: GTTAAATCTGGACCCACTTGCC R: GGGTGAATTAGTGAACGTCTTG	161–180	7	0.805 ± 0.125	0.718 ± 0.074
13-N1132836	(AAG)7	MT515710	F: GTTCCTGCTCCGAGACCTAGAA R: TCATGAAGAAATCGCAACCA	138–144	3	0.304 ± 0.087	0.296 ± 0.086
16-N1150626	(TGG)5	MT515711	F. GTCTTTGGCGAAATCAGTTGGT R: GAAGGTTTCCAGGTTGGTGA	149–159	3	0.299 ± 0.186	0.249 ± 0.109
18-N1173706	(AAG)6	MT515712	F: GAGCTGCCTCGATATGGACT R: GTTATCCAATGGCCAAGAAACC	164–170	3	0.398 ± 0.105	0.421 ± 0.078
31-N2284857	(TTC)12	MT515713	F: GTCTCTTGCTATCATGCGGTCA R: CAGATTGGAGAATCCAGACCA	207–263	9	0.771 ± 0.138	0.812 ± 0.078
33-N3477883	(TGA)6	MT515714	F: GTACAAGATTCATGTGACCGGC R: GCAAGGCATCATATTCACGA	184–200	4	0.550 ± 0.171	0.472 ± 0.124
43-N1009973	(AT)10	MT515715	F: GTTGCGCTCATCGATCTGTA R: GTGCGAACTATGATCGGACGAA	146–185	9	0.439 ± 0.102	0.778 ± 0.027

For each loci, the repeat motif, Genbank accession number, primer sequence, and size range (bp) is indicated. *Na* refers to the number of alleles, *Ho* to observed heterozygosity (mean ± SE) and *He* to expected heterozygosity (mean ± SE).

From the three sampling areas of *W. salutaris* 156 alleles were found in the 48 individuals sampled, being the number of alleles higher in LM than in the other two areas (Table 2). The average Shannon’s diversity index (*I*) was also higher in LM than in TR and FC. Observed and expected heterozygosis had similar average values in LM and TR being slightly lower in FC. The polymorphic information content (*PIC*) had high average values while inbreeding coefficients (*F*_*IS*_) were low and showing negative values in the three sampling areas.

**Table 2:** Genetic diversity of *Warburgia salutaris* in the three study areas.

Locus	Lebombo Mountains (LM)					Tembe River (TR)					Futi Corridor (FC)				
	*Na*	*I*	*Ho*	*He*	*PIC*	*Na*	*I*	*Ho*	*He*	*PIC*	*Na*	*I*	*Ho*	*He*	*PIC*
1-N1002135	3	0.809	0.579	0.499	0.499	3	0.840	0.667	0.491	0.491	2	0.340	0.214	0.191	0.191
7-N1082598	7	1.457	0.526	0.672	0.672	9	1.764	0.800	0.758	0.758	8	1.516	0.571	0.671	0.671
10-N1110523	9	1.942	0.895	0.832	0.832	5	1.480	0.733	0.762	0.762	7	1.649	0.929	0.763	0.763
12-N1126672	7	1.716	0.842	0.795	0.795	7	1.739	1.000	0.789	0.789	4	1.061	0.571	0.569	0.569
13-N1132836	2	0.576	0.421	0.388	0.388	2	0.245	0.133	0.124	0.124	2	0.562	0.357	0.375	0.375
16-N1150626	3	0.455	0.158	0.234	0.234	2	0.637	0.667	0.444	0.444	2	0.154	0.071	0.069	0.069
18-N1173706	3	0.942	0.579	0.564	0.564	3	0.680	0.400	0.407	0.407	2	0.469	0.214	0.293	0.293
31-N2284857	14	2.429	0.947	0.895	0.895	9	1.884	0.867	0.816	0.816	6	1.487	0.500	0.727	0.727
33-N3477883	4	0.954	0.526	0.517	0.517	3	0.468	0.267	0.238	0.238	3	1.090	0.857	0.661	0.661
43-N1009973	11	1.980	0.632	0.801	0.810	9	1.827	0.400	0.798	0.798	5	1.438	0.286	0.724	0.724
Average ± SE	6.300 ± 1.274	1.326 ± 0.213	0.611 ± 0.075	0.620 ± 0.068	0.621 ± 0.216	5.200 ± 0.952	1.156 ± 0.203	0.593 ± 0.089	0.563 ± 0.081	0.563 ± 0.256	4.100 ± 0.722	0.977 ± 0.175	0.457 ± 0.089	0.504 ± 0.080	0.504 ± 0.252

*Na* refers to the number of alleles, *I* to Shannonʼs diversity index, *Ho* to observed heterozygosity (mean ± SE), *He* to expected heterozygosity (mean ± SE) and *PIC* to polymorphic information content.

### Population genetic structure and differentiation

**Figure 2:**
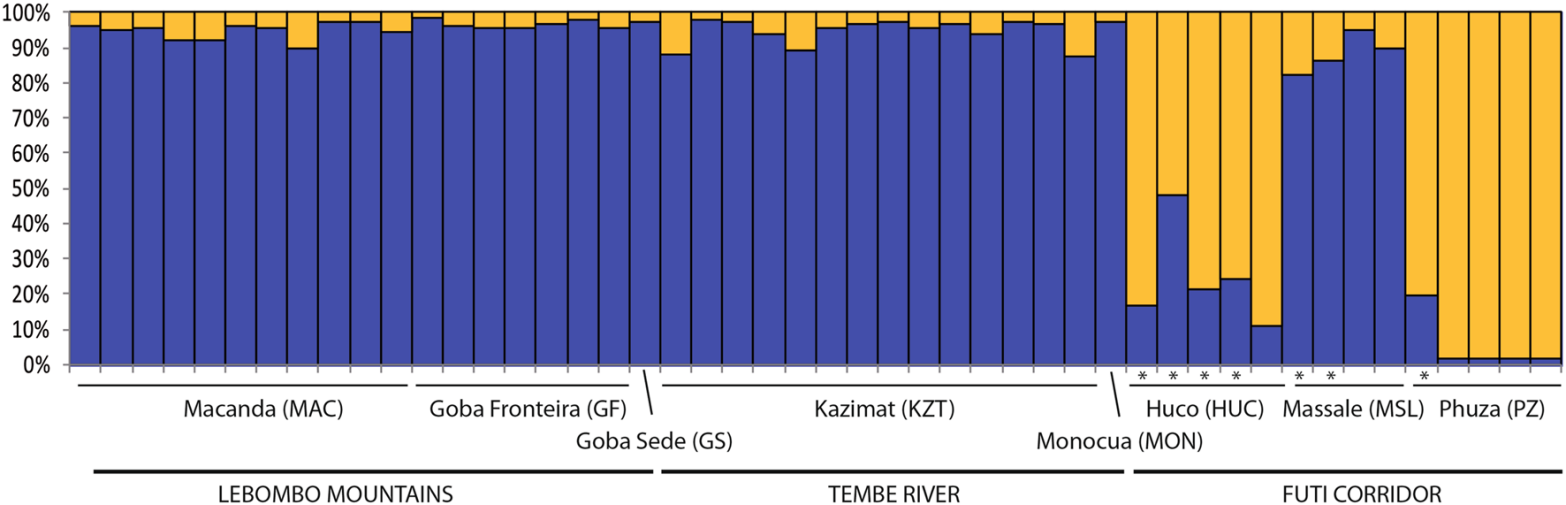
Population structure of *Warburgia salutaris* based on 10 SSRs and using the best assignment result retrieved by STRUCTURE (*K* = 2). Each individual sample is represented by a thin vertical line divided into *K* coloured segments that represent the individual’s estimated membership fractions in *K* clusters. Populations and main geographical areas are indicated below following Table 4. Asterisks indicate individuals with a probably of membership lower than 90% to the main genetic cluster, as revealed by STRUCTURE.

The Bayesian clustering program STRUCTURE found the highest LnP(D) and ΔK values for *K* = 2 (Fig. 2; Fig. S1). One cluster was predominantly found across LM and TR areas, while a second one characterized the FC area. Nevertheless, some individuals in this last area showed signs of genetic admixture between the two genetic groups (* indicated in Fig. 2).

**Figure 3:**
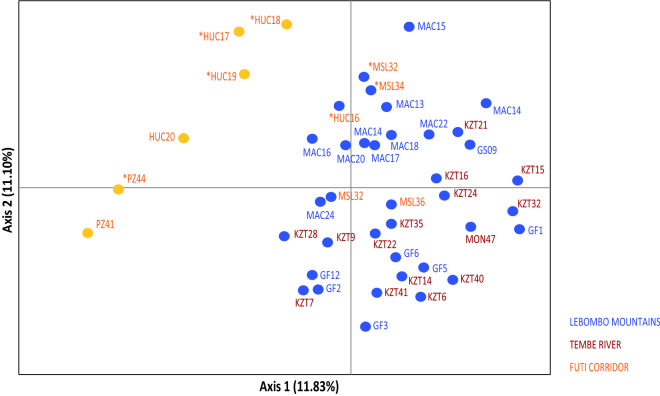
Principal Coordinate analysis (PCoA) of the studied *Warburgia salutaris* using the scored SSRs markers. Percentage of explained variance of each axis is given in parentheses. Population labels follow Table 4. Colour of symbols (circles) indicate the two genetic groups identified by STRUCTURE. Colour of labels follow the three main geographic areas as depicted in Fig. 1. Asterisks as in Fig. 2.

**Figure 4:**
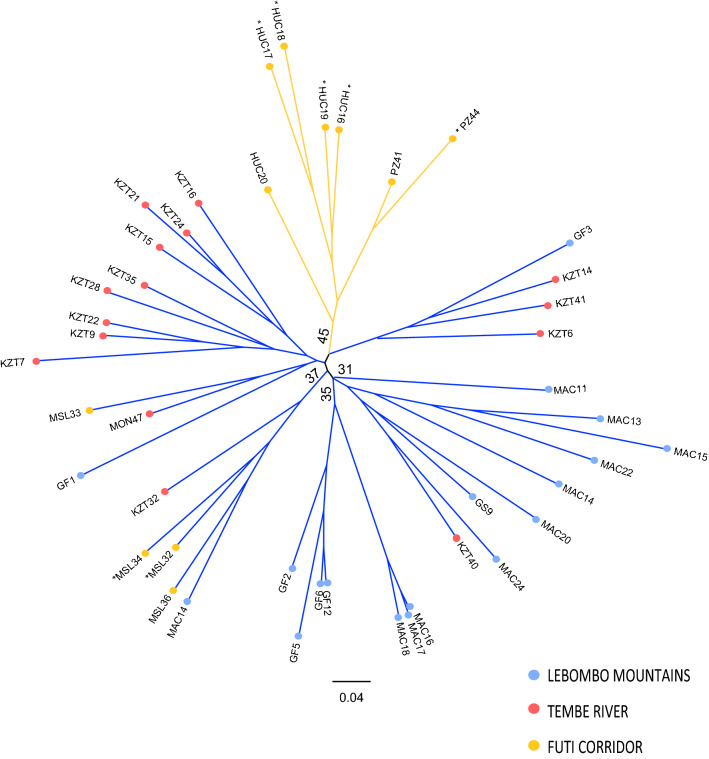
Unrooted neighbour-joining tree of the studied *Warburgia salutaris* based on Nei’s Da genetic distance. Numbers associated with branches indicate bootstrap values (BS) based on 1000 replications. Only BS above 30 are shown. Colours of branches indicate the two genetic groups identified by STRUCTURE. Colour of circles near each label indicate the three main geographic areas as depicted in Fig. 1. Asterisks as in Fig. 2.

The first two coordinates of the principal coordinate analysis (PCoA) explained 22.9% of the total variation, and populations were spatially separated into the two main groups found by STRUCTURE (Fig. 3). The neighbour-joining tree revealed several small clusters although mostly with a very low support (< 30% BS) and overall, with no association between the clusters found and the three geographic areas (Fig. 4) as reported in the other analyses. However, a clear cluster grouped all the FC geographical area.

The pairwise population *F*_ST_ values varied from 0.049 (TR vs. LM) to 0.114 (FC vs. TR) revealing moderate levels of genetic differentiation between FC and TR and between FC and LM and lower levels between TR and LM (Table 3).

**Table 3:** Pairwise population *F*_ST_ values for *Warburgia salutaris* in the three study areas.

Population	Lebombo Mountains	Tembe River	Futi Corridor
Lebombo Mountains	0.000		
Tembe River	0.049	0.000	
Futi Corridor	0.084	0.114	0.000

## Discussion

### High genetic diversity and admixture in *Warburgia salutaris*

Assessment of genetic diversity is critical to understand the ability of a species to cope with changing conditions and environments, especially for threatened species^39,64–68^. In this study, we reported for the first time the development of Single Sequence Repeats (SSR) markers in *W. salutaris* by employing next generation sequencing (Illumina platform). The 10 SSRs markers were validated and found to be highly polymorphic, with values similar to the ones found in other threatened species such as *Acer miaotaiense* (*PIC* = 0.604)^69^ or *Corylus avellana* (*PIC* = 0.778)^70^. These markers are now available to extend *W. salutaris* population studies to a worldwide level. Additionally, the SSRs developed during this work might potentially be suitable to study genetic diversity in other species within the genus *Warburgia*, since only a limited number of studies is available and based on Amplified Fragment Length Polymorphism (AFLP)^62,71^, a time-consuming and costly technique. To the best of our knowledge, the present study represents the first genome size estimation of *W. salutaris* and only the second within the Canellaceae family having a genome size 4 × smaller than *Canella winterana* (2C = 11.7 pg^72,73^). The relatively small genome size of *W. salutaris* (see methods) is within the range of the non-expanded genomes of currently known magnoliids (Fig. S2) and may facilitate future genomic initiatives although further analyses are needed to determine its ploidy level.

Due to the heavy harvesting pressure to which *W. salutaris* is subjected in Mozambique^28,48^, genetic diversity levels were expected to be low. However, we found high levels of genetic diversity in the three surveyed areas in comparison to other narrowly distributed species, as for instance, the tropical tree *Paypayrola blanchetiana* (*Na:* 2–5 alleles per locus; *Ho:* 0.063–0.563 in the two populations; *He:* 0.063–0.567 in the first population and 0.063–0.627 in the second)^39^. However, genetic diversity indices of *W. salutaris* were similar to other species where bark has been heavily-exploited, such as *Cinchona officinalis* (*Na:* 5.2–7.6 alleles per locus; *Ho:* 0.580–0.680; *He:* 0.616–0.717)^74^ or even lower than *Himatanthus drasticus* (*Na:* 6–24; *Ho*: 0–0.847, *He*: 0–0.864)^34^.

High levels of heterozygosis may be due to factors including the reproductive system such as self-incompatibility^75^ or high gene flow^65,76^. Results from this work revealed a range of the inbreeding coefficient of -0.492 (TR) to -0.363 (LM), which is much lower than those found in *e.g. H. drasticus* (0.248–0.303)^34^, *Calotropis gigantea* (0.167), *C. procera* (0.177)^77^, or *Phoenix theophrasti* (0.9)^78^. The negative inbreeding values found here suggest the existence of random mating^79^ among individuals of *W. salutaris* and might also explain the levels of heterozygosis found here. Indeed, the related species *Warburgia ugandensis* has a mixed mating system being predominantly outcrossing^62^. Additionally, insect pollinators of *W. salutaris* such as bees are probably able to travel over the large agricultural blocks separating the three geographical areas studied here, promoting gene flow. Genetic admixture between sites might also be facilitated by frugivorous birds that often eat the berries thereby facilitating the dispersion of seeds. In accordance, we found high levels of genetic admixture between populations with only two genetic clusters being found, one grouping the northern populations and the other one, the southern populations.

Our study suggests that, although some local populations might have been severely affected by harvesting, the pepper-bark tree might have persisted in sufficient numbers in Mozambique to allow outcrossing between sites, retaining a large proportion of genetic diversity. Although there are no records of the historical distribution of this species, the studied populations could be relicts of once much larger populations that persisted in specific locations. In addition, recent conservation efforts might have diminished trade in Mozambique, avoiding severe barking in these populations. Further research should focus on understanding the factors limiting the regeneration of *W. salutaris* trees.

### Population differentiation between geographic areas

Population differentiation of endangered species is variable. For example, low differentiation was found between populations of *Platanthera leucophaea* (F_ST_ < 0.02 over distances < 2 km^80^) while in *H. drasticus* the differentiation levels were high (F_ST_ from 0.036 to 0.077 over short distances)^34^. In contrast, the endangered *Paeoma rockii* revealed a high differentiation between populations (F_ST_ varied from 0.780 to 0.982)^81^. Despite the narrow distributional area of *W. salutaris* in Mozambique, this study revealed a high genetic differentiation between the northern populations located in LM and TR and the southern populations located in FC (Fig. 1). Pairwise F_ST_ comparisons showed lower genetic differentiation between LM and TR (0.049), which are separated by only 28 km, than either between LM and FC areas (0.084, separated by 81 km) or between TR and FC (0.114, separated by 49 km). STRUCTURE analyses also found a distinct genetic cluster in the FC area, which was also supported by PCoA analyses and the NJ tree. Contrary to LM and TR areas, where *W. salutaris* occurs in slopes and forest patches, in the FC area this species occurs near seasonal pans in thicket vegetation associated with termitaria on clay soils^82,83^. This might imply differences in reproductive ecology, particularly regarding flowering phenology and the activity of pollinators, which would affect gene flow with the other sites, explaining the genetic structure and population differentiation found between the studied sites. Thus, the differentiated FC genetic clusters could be harbouring novel and important alleles and should be given priority in in situ and ex situ conservation strategies in Mozambique^77,84,85^.

### How to conserve a species widely exploited and needed?

Several populations of *W. salutaris* are threatened by fire from slash and burn agriculture, as they occur in adjacent patches or in agricultural lands^48^. Equally, burning of natural vegetation to improve livestock fodder, poaching, and opening of new areas for settlements are also potential threats to the species (e.g.^86–88^). Vegetative propagation of *W. salutaris* is possible through tissue culture^63^ although expensive. This species is being largely cultivated ex situ in South Africa^89^ and in small scale in Zimbabwe^53^ and Mozambique (unpublished data), to encourage the sustainable use of the species. Home gardening would also be important for this species although that requires the involvement of local communities and understanding their perceptions towards the conservation of this species.

Considering the confined distribution and threatened status, the long-term persistence of *W. salutaris* should be secured by conserving the maximum genetic diversity of the species. As it is impossible to designate every natural wild plant habitat as a protected area, nurseries could be implemented to ensure production stability. The disclosure of genetic variation and understanding of genetic relatedness within populations is useful for their sustainable uses^90^. Knowledge of genetic diversity from other countries as the one reported here would also help to implement conservation strategies including re-introduction programs, selecting the most suitable material to be used. Understanding the degree of genetic variation between Mozambique and the neighbouring countries would facilitate transborder conservation actions. Further studies must also be conducted to detect and understand how reductions of natural regeneration or fitness are affected by harvesting. Finally, efforts to educate the local population and landowners on the importance of conserving the natural populations of *W. salutaris* should continue.

## Methods

### Study species

*Warburgia salutaris* is an evergreen tree, generally 5–10 m tall, but occasionally up to 20 m^57^. The flowers are small (< 7 mm in diameter), white to greenish in colour, generally solitary or in tight, few-flowered heads, borne on short, robust stalks in the axils of the leaves from autumn to winter (April–June). Flowers are bisexual, actinomorphic (having symmetrically arranged perianth parts of similar size or shape that are divisible into 3 or more equal halves). Flowers are visited by many insect species, most especially bees. The flowers develop rounded, oval berries (30 mm in diameter), usually dark-green and turning purple during ripening that occurs throughout winter an into early summer (July to December). Dispersion occurs by frugivorous birds that disperse the seeds, although fruits can also drop near the maternal tree. Leaves are glossy and dark green, with a bitter, peppery taste. The stem is covered by a brown bark marked with corky lenticels and is bitter and peppery and is widely used medicinally. The active compounds (drimanes and sesquiterpenoides) are mostly found in the inner part of the stem and root bark.

### Study area

The present study was carried out in the districts of Matutuine and Namaacha (Mozambique), in the three areas of known occurrence of *W. salutaris*^48^: (1) Lebombo Mountains (LM) also named the western area, (2) Tembe River (TR) or centre, and (3) Futi Corridor (FC) or eastern area (Fig. 1). The climate is subtropical to tropical, encompassing a wet (October–April) and dry season (May–September). The mean annual temperature ranges from 21 to over 24 °C, and the mean annual rainfall from 600 to 1000 mm^88,91^. In LM, *W. salutaris* is accompanied by *Acacia nigrescens* Oliv., *Acacia burkei* Benth. and *Combretum apiculatum* Sond, although *Aloe marlothii* A. Berger, *Ficus* spp. and *Euphorbia* spp. are found in shallow soils, and *Olea africana* Miller and *Combretum* spp. in steeper stony slopes^92,93^. In TR, *W. salutaris* is found in sand forest patches together with *Pteleopsis myrtifolia* (M.A.Lawson) Engl. & Diels, *Cleistanthus schlechteri* Pax (Hutch.), *Hymenocardia ulmoides* Oliv. and *Monodora junodii* Engl. & Diels^94^. The open savanna woodland links the patches of *W. salutaris*, being composed mainly by *Strychnos* spp., *Terminalia sericea* Burch. ex DC., *Acacia burkei* Benth., *Combretum molle* R. Br. ex G.Don and *Albizia versicolor* Oliv.^95^. In FC, *W. salutaris* occurs near seasonal pans^96^ in thicket vegetation associated to termitaria in clay soils^82^. Common tree species found in this community include *Berchemia zeyheri* (Sond.) Grubov, *Pappea capensis* Eckl. & Zeyh. and *Olea europaea* subsp. *africana* (Miller) P.S. Green^97^. The primary economic activities of local residents are subsistence agriculture, livestock rearing, trade of non-timber forest products and migrant labour to South Africa^87,98,99^.

### Population sampling, DNA extraction, genome size value, and SSR development

Based on the areas of occurrence (Senkoro et al., unpublished data), 48 individuals were sampled: 19 individuals from LM, 15 from TR and 14 from LM (Table 4). Fresh, young undamaged leaves were collected for each individual plant and frozen at − 80 °C until DNA isolation. Total genomic DNA was extracted from 50 mg of ground leaves using the InnuSPEED Plant DNA Kit (Analytik Jena Innuscreen GmbH, Germany) according to the manufacturer’s protocol. The average yield and purity were assessed spectrophotometrically by OD230, OD260 and OD280 readings (Nanodrop 2000, Thermo Fisher Scientific, Waltham, MA, USA) and visualized by electrophoresis in 1% agarose gels under UV light. Normalized DNA from five individuals of each population was used to develop the SSR markers at CD Genomics (cd-genomics.com/hi-ssrseq.html).

**Table 4:** Sampled accessions and locations of *Warburgia salutaris* sorted by geographical area.

Accessions	Location	ID	Lat	Long	Accessions	Location	ID	Lat	Long
GF1 (1)	Goba Fronteira	LM	− 26.23266	32.09810	KZT16 (27)	Kazimat	TR	− 26.40994	32.35490
MAC13 (2)	Macanda	LM	− 26.03522	32.12181	KZT21 (28)	Kazimat	TR	− 26.40391	32.36711
MAC14 (3)	Macanda	LM	− 26.03577	32.12150	KZT22 (29)	Kazimat	TR	− 26.40059	32.35109
MAC15 (4)	Macanda	LM	− 26.03778	32.12730	KZT24 (30)	Kazimat	TR	− 26.40206	32.36188
MAC16 (5)	Macanda	LM	− 26.03692	32.12772	KZT28 (31)	Kazimat	TR	− 26.36735	32.37323
MAC17 (6)	Macanda	LM	− 26.05158	32.11803	KZT35 (32)	Kazimat	TR	26.36737	32.37266
MAC18 (7)	Macanda	LM	− 26.05159	32.11565	KZT40 (33)	Kazimat	TR	− 26.36873	32.37078
MAC19 (8)	Macanda	LM	− 26.81118	32.64545	KZT41 (34)	Kazimat	TR	− 26.36929	32.37334
MAC20 (9)	Macanda	LM	− 26.04696	32.11979	KZT46 (35)	Kazimat	TR	− 26.36935	32.37321
MAC22 (10)	Macanda	LM	− 26.04508	32.11982	MON47 (36)	Monucua	TR	− 26.36952	32.32288
MAC24 (11)	Macanda	LM	− 26.03521	32.12181	Huc16 (44)	Huco	FC	− 26.85013	32.60338
GF2 (12)	Goba Fronteira	LM	− 26.26867	32.10719	Huc17 (45)	Huco	FC	− 26.86159	32.60604
GF5 (13)	Goba Fronteira	LM	− 26.23250	32.09818	Huc18 (46)	Huco	FC	− 26.86169	32.60353
GF6 (14)	Goba Fronteira	LM	− 26.23241	32.09815	Huc19 (47)	Huco	FC	− 26.86129	32.60282
GF12 (15)	Goba Fronteira	LM	− 26.23240	32.09822	Huc20 (48)	Huco	FC	− 26.86025	32.60309
GS09 (16)	Goba Sede	LM	− 26.23238	32.09822	MSL32 (49)	Massale	FC	− 26.83979	32.88339
MAC11 (17)	Macanda	LM	− 26.04509	32.11983	MSL33 (50)	Massale	FC	− 26.86458	32.60790
GF3 (18)	Goba Fronteira	LM	− 26.26879	32.10747	MSL34 (51)	Massale	FC	− 26.80948	32.64368
GF4 (19)	Goba Fronteira	LM	− 26.23233	32.09818	MSL36 (52)	Massale	FC	− 26.80590	32.63823
KZT6 (22)	Kazimat	TR	− 26.41303	32.36338	Pz41 (53)	Phuza	FC	− 26.78824	32.67368
KZT7 (23)	Kazimat	TR	− 26.41190	32.36422	Pz42 (54)	Phuza	FC	− 26.78817	32.67434
KZT9 (24)	Kazimat	TR	− 26.40960	32.36578	Pz43 (55)	Phuza	FC	− 26.78814	32.67383
KZT14 (25)	Kazimat	TR	− 26.40414	32.35073	Pz44 (56)	Phuza	FC	− 26.78760	32.67419
KZT15 (26)	Kazimat	TR	− 26.38806	32.35008	Pz45 (57)	Phuza	FC	− 26.81144	32.66415

*LM* Lebombo Mountains, *TR* Tembe River, *FC* Futi Corridor.

For the development of the markers, we first estimated the nuclear DNA content of *W. salutaris* by flow cytometry using fresh young leaves that were chopped using a razor blade together with an internal standard in a Petri dish containing 1 mL of Woody Plant Buffer^100^ following the protocol described in^101^. *Solanum lycopersicum* ‘Stupické’ (2C = 1.96 pg)^102^ was used as internal standard. The nuclear suspension was then filtered through a 30 μm nylon filter, and 50 μg/mL of propidium iodide (PI; Sigma-Aldrich, St. Louis, USA) and 50 μg/mL of RNase (Sigma-Aldrich) were added to stain the DNA only. The fluorescence intensity of nuclei was analysed using a CyFlow Space flow cytometer (Sysmex, Kobe, Japan). Four independent replicates collected from Kazimat (TR) were measured. Conversion of mass values into numbers of base pairs was done according to the factor 1 pg = 978 Mbp^103^. The mean 2C-value of *W. salutaris* was found to be 2.91 pg (± 0.068), corresponding to an average genome size of 2845 Mbp (Fig. S2). Samples had an average coefficient of variation of 4.18%.

Genomic libraries were constructed using the KAPAHyper prep kit and sequenced by Illumina Hiseq 2500. We firstly used SSRHunter1.3 to screen the potential SSRs from the sequenced data that had at least five repeats (penta-) for 3–5 bp units. Based on the obtained sequences, primers were designed with Primer Premier 5.0 software (Table 1). Fourteen geographically representative samples of *W. salutaris* (LM, TR and FC; Fig. 1) were first used to test microsatellite amplification and to troubleshoot amplification conditions. Amplifications were performed in 15 μl reactions containing: 1.25U TaKaRa Hot startTaq polymerase, 1X Buffer I, 1 mM dNTPs, 5 μM Primer F and R and 100 ng DNA. The PCR amplification conditions were run as follows: 95 °C for 5 min, 94 °C for 30 s, 30 cycles of 56 °C for 30 s, 72 °C for 30 s, 94 °C for 30 s, 10 cycles of 53 °C for 30 s, 72 °C for 30 s and final extension at 60 °C for 30 min. We then considered 10 markers that presented > 20% polymorphism, which were used to amplify all samples within this study (Table 1). The amplified fragments were analysed on a 3730 × 1 gene analyzer (Thermo Fischer Scientific) and examined manually for microsatellite peaks. Allele sizes were determined using GeneMapper 3.2 (Applied Biosystems).

### Estimates of genetic diversity

For each microsatellite locus, genetic polymorphism was assessed in 48 individuals by calculating the number of alleles (*Na*), observed heterozygosis *(Ho*), expected heterozygosis (*He*), Shannon’s diversity index (*I*), and inbreeding coefficient (*F*_*IS*_) using GenALEX software version 6.5^104^. The polymorphic information content (*PIC*) was calculated as *PIC* = 1 − ΣP_i_^2^, where P_i_ is the allele frequency for each SSR marker locus^105,106^. Values of *PIC* above 0.5 were considered highly informative, between 0.5 and 0.25 moderately informative, and below 0.25 less informative^107^.

### Population genetic structure and differentiation

The Bayesian program STRUCTURE v.2.3.4^108^ was used to infer the population structure and to assign individual plants to subpopulations. Models with a putative numbers of populations (K) from 1–5, imposing ancestral admixture and correlated allele frequencies priors, were considered. Ten independent runs with 50 000 burn-in steps, followed by run lengths of 1 000 000 interactions for each K, were computed. The number of clusters in the data was estimated using STRUCTURE HARVESTER^109^, which identifies the optimal K based both on the posterior probability of the data for a given K and the ΔK^110^. To correctly assess the membership proportions (q values) for clusters identified in STRUCTURE, the results of the replicates at the best fit K were post-processed using CLUMPP 1.1.2^111^. GenALEX software version 6.5^104^ was used to calculate the Nei’s genetic distance^112^ among individuals. A Principle Coordinate Analysis (PCoA)^113^ was performed to detect genetic variations between *W. salutaris* individuals. POPULATION 1.2^114^ was used to construct an unrooted neighbour-joining tree with 1000 bootstrap replicates. The Wright’s F_ST_ value was computed to estimate population differentiation^104^. Lower genetic differentiation was considered for F_ST_ below 0.05, moderate from 0.05 to 0.15 and high genetic differentiation above 0.25^115^.

## Supplementary information

Supplementary infomation.

Supplementary infomation

Supplementary infomation
