# Supplementary material for: The genetic legacy of fragmentation and overexploitation in the threatened medicinal African pepper-bark tree, Warburgia salutaris
Source: Sci Rep. 2020 Nov 12;10:19725. doi: 10.1038/s41598-020-76654-6 (PMC7661512; doi:10.1038/s41598-020-76654-6)

The genetic legacy of fragmentation and overexploitation in the threatened medicinal African pepper-bark tree, Warburgia salutaris

Annae M. Senkoro1,2, Pedro Talhinhas3, Fernanda Simões4, Paula Batista-Santos3, Charlie M. Shackleton1, Robert A. Voeks5, Isabel Marques6, Ana I. Ribeiro-Barros6
1 Department of Environmental Science, Rhodes University, Grahamstown 6140, South Africa
2 Departmento de Ciências Biológicas, Universidade Eduardo Mondlane CP 257, Maputo Moçambique
3 Linking Landscape, Environment, Agriculture and Food (LEAF), Instituto Superior de Agronomia, Universidade de Lisboa, Tapada da Ajuda, 1349-017 Lisbon, Portugal
4 Instituto Nacional de Investigação Agrária e Veterinária, Av. da República,Quinta Marquês, Edificio Sede, 2780-157 Oeiras, Portugal
5 Department of Geography & the Environment, California State University, Fullerton, 800 N State College Blvd, Fullerton, CA 92831 USA
6 Forest Research Centre (CEF), Instituto Superior de Agronomia, Universidade de Lisboa, Tapada da Ajuda, 1349-017 Lisbon, Portugal

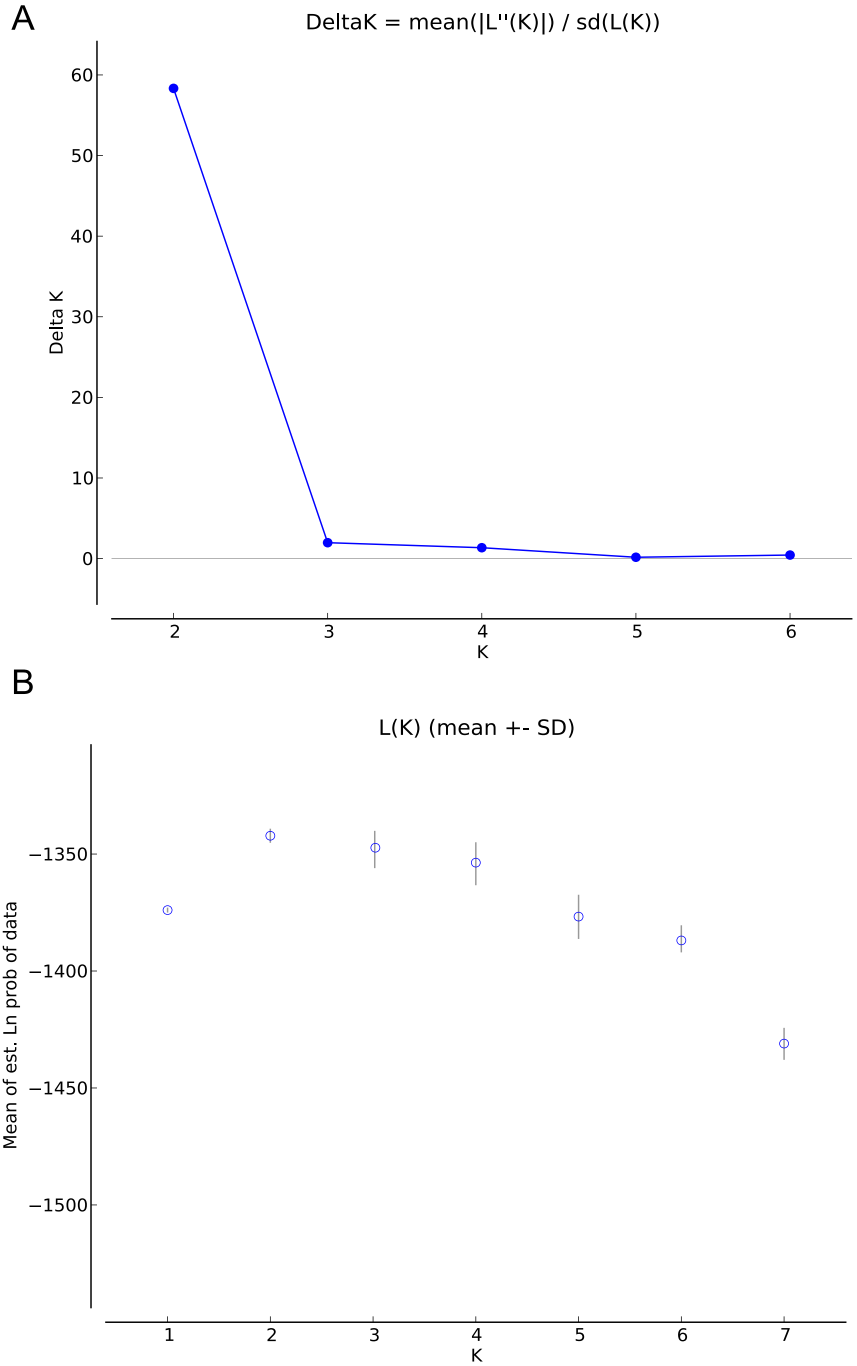

Supplement: Supplementary file 1 — Supplementary infomation. [file 41598_2020_76654_MOESM1_ESM.pdf]
